# Air pollution, life’s essential 8, and risk of severe non-alcoholic fatty liver disease among individuals with type 2 diabetes

**DOI:** 10.1186/s12889-024-18641-4

**Published:** 2024-05-20

**Authors:** Ruxianguli Aimuzi, Zhilan Xie, Yimin Qu, Yu Jiang

**Affiliations:** School of Population Medicine and Public Health, Chinese Academy of Medical Sciences and Peking Union Medical College, Beijing, 100730 China

**Keywords:** Non-alcoholic fatty liver disease (NAFLD), Air pollution, Life’s Essential 8 (LE8), Type 2 diabetes (T2D)

## Abstract

**Background:**

The impacts of long-term exposure to air pollution on the risk of subsequent non-alcoholic fatty liver disease (NAFLD) among participants with type 2 diabetes (T2D) is ambiguous. The modifying role of Life’s Essential 8 (LE8) remains unknown.

**Methods:**

This study included 23,129 participants with T2D at baseline from the UK Biobank. Annual means of nitrogen dioxide (NO_2_), nitrogen oxides (NO_X_), and particulate matter (PM_2.5_, PM_2.5–10_, PM_10_) were estimated using the land-use regression model for each participant. The associations between exposure to air pollution and the risk of severe NAFLD were evaluated using Cox proportional hazard models. The effect modification of LE8 was assessed through stratified analyses.

**Results:**

During a median 13.6 years of follow-up, a total of 1,123 severe NAFLD cases occurred. After fully adjusting for potential covariates, higher levels of PM_2.5_ (hazard ratio [HR] = 1.12, 95%CI:1.02, 1.23 per interquartile range [IQR] increment), NO_2_ (HR = 1.15, 95%CI:1.04, 1.27), and NO_X_ (HR = 1.08, 95%CI:1.01, 1.17) were associated with an elevated risk of severe NAFLD. In addition, LE8 score was negatively associated with the risk of NAFLD (HR = 0.97, 95% CI: 0.97, 0.98 per point increment). Compared with those who had low air pollution and high LE8, participants with a high air pollution exposure and low LE8 had a significantly higher risk of severe NAFLD.

**Conclusions:**

Our findings suggest that long-term exposure to air pollution was associated with an elevated risk of severe NAFLD among participants with T2D. A lower LE8 may increase the adverse impacts of air pollution on NAFLD.

**Supplementary Information:**

The online version contains supplementary material available at 10.1186/s12889-024-18641-4.

## Introduction

Non-alcoholic fatty liver disease (NAFLD) is characterized by the buildup of hepatic fat in the liver (≥ 5%) without notable indications of excessive alcohol use, hepatic viral infection, or chronic liver disease [1, 2]. NAFLD currently affects more than 25% of people worldwide and commonly coexists with type 2 diabetes (T2D), obesity, metabolic dysregulation, and other factors [3, 4]. Among these comorbidities, T2D significantly contributes to the pathogenesis of NAFLD and the associated burden of the disease [5]. Compared to non-T2D individuals, people with T2D are at a higher risk of developing NAFLD, hepatocellular carcinoma, and liver-related mortality [5–7]. Thus, deciphering modifiable factors affecting NAFLD in individuals with T2D is critical to the prevention and management of NAFLD [1, 8, 9].

Air population has been recognized as a major global health risk factor [10]. Recent epidemiological studies have reported that long-term exposure to ambient air pollutants, such as particulate matter (PM) with diameters ≤ 2.5 μm (PM_2.5_) and nitrogen dioxide (NO_2_), was associated with an elevated risk of NAFLD [11–14]. Although the mechanisms are only partially understood, air pollution-induced inflammation, oxidative stress, and endothelial function, which are also the basis for the development of T2D related to air pollution exposure, have been implicated in the pathogenesis of NAFLD [15]. Given the complex link between NAFLD and T2D, individuals with T2D may be more susceptible to the adverse effects of air pollution on NAFLD [16, 17]. However, epidemiological evidence directly linking air pollution to the risk of NAFLD among this susceptible population is still very limited.

In addition to environmental factors (e.g., air pollution), unhealthy lifestyles and unfavorable metabolic status play fundamental roles in the development of many interconnected metabolic diseases, including NAFLD [18, 19]. Recently, the Life’s Essential 8 (LE8), a composite index encompassing four lifestyle factors (i.e., nicotine exposure, physical activity, sleep health, diet) and four metabolic factors (body mass index [BMI], blood pressure, blood lipid status, and glucose level) have been proposed to better prevention and management of cardiometabolic diseases [20]. Notably, a lower LE8 has been related to the increased risk of NAFLD [21, 22]. However, whether LE8 could modify the association between air pollution and the risk of NAFLD has not been explored, especially among individuals with T2D.

To fill these gaps, the present study aimed to explore the associations of air pollutants with severe NAFLD among T2D participants and assess the interaction between LE8 and air pollution in a large prospective cohort study.

## Methods

### Study population

The UK Biobank study is an ongoing prospective cohort study with more than 0.5 million adults (aged 40–69 years) recruited from 22 centers throughout the United Kingdom from 2006 to 2010 [23]. For the present study, a total of 26,836 individuals with prevalent T2D at baseline were identified using algorithms introduced by a previous study [24]. Specifically, prevalent T2D was defined based on a combination of self-reported medical history, abnormal glucose level and glycated hemoglobin (HbA1c) level at baseline, and hospital inpatient records. Self-reported medical history was collected using a touchscreen questionnaire and verbal interview conducted by trained staff at the baseline assessment. T2D patients were also identified based on random glucose levels ≥ 11.1 mmol/L or HbA1c levels ≥ 48 mmol/mol [24]. For those who do not provide responses to the above questions or without available laboratory markers, information from the hospital inpatient records was used (Fig. S1). After the exclusion of those with missing data on air pollution, adjusted covariates (i.e., race, Townsend Deprivation Index [TDI] score, and education level), and prevalent liver-relevant diseases (e.g., alcoholic liver disease, alcohol abuse, NAFLD, cirrhosis, liver failure, etc. at/before baseline, see Table S1 for details), a total of 23,129 participants were finally included in primary association analyses of air pollution with severe NAFLD.

### Exposure assessment

Annual average concentrations of ambient air pollution, including PM_2.5_, PM_10_, PM_2.5–10_, NO_2_, and nitrogen oxides (NO_X_), were estimated using land use regression (LUR) models according to the home address of participants collected at baseline recruitment [25]. LUR models were conducted with predictor variables, including land use, population density, and traffic, obtained from the Geographical Information System [25, 26]. The median model explained variances (R^2^) of the LUR models for PM_2.5_, PM_10_, NO_2_, and NO_X_ ranged from 71%-82% [25, 26]. Data on PM_2.5_, PM_2.5–10_, and NO_X_ levels were available only in 2010, while data on NO_2_ level was available in 2005–2007 and 2010, and data on PM_10_ was available in 2007 and 2010. Levels of pollutants available for multiple years were averaged. Meanwhile, a weighted air pollution score was established to evaluate the overall exposure to various air pollutants using the following equation: $\sum {\left(\upbeta {\left[i\right]}^{*} i\right)}^{*} 5 / \sum\upbeta \left[i\right]$ and i referred to air pollutants [27].

### Ascertainment of severe NAFLD

The outcome of this study is severe NAFLD, which was obtained via links to the hospital and death record. NAFLD was identified with International Classification of Diseases-10 (ICD-10) codes K75.8 and K76.0 according to previous studies [21, 28, 29] (see Table S2 for details).

### Assessment of LE8

The complete quantitative evaluation of LE8 has been previously documented [30] and presented in Table S3-S4. The constituents of LE8 include four health behaviors (diet, physical activity, tobacco/nicotine exposure, sleep health) and four metabolic factors (BMI, blood lipids, glucose, and blood pressure). The healthy diet score was calculated based on the information collected at baseline using the Food Frequency Questionnaire (Table S4). Information on physical activity (minutes of moderate or vigorous physical activity per week), tobacco/nicotine exposure (tobacco use or secondhand smoke exposure), and sleep health (average hours of sleep per night) were obtained from self-reported questionnaires at baseline. BMI and blood pressure (average of all available blood pressure measurements) were measured at baseline recruitment. The glucose score was evaluated using HbA1c, whereas the lipid score was assessed using non-high-density lipoprotein cholesterol. The scoring for each metric of LE8 spans a range from 0 to 100, with higher scores indicating healthier health-related behaviors and factors [30]. Overall LE8 was computed as the average of 8 components, with the final value ranging from 0 to 100.

### Statistical analysis

Cox proportional hazard models were used to evaluate the association of air pollutants with severe NAFLD. Schoenfeld residual methods were used to examine proportional hazard assumptions, and no noticeable indication of a violation was observed in the present study. The air pollutants were modeled as continuous (per IQR increase in each air pollutant) and categorical (quartiles) variables. Linear trend tests by the quartiles of air pollutants were evaluated using the ordinal variables in the Cox proportional hazard models. The follow-up time was calculated from the date of baseline recruitment to the date of first diagnosis of severe NAFLD, death, loss to follow-up, or censor (31 March 2023), whichever occurred first.

We considered three adjustment models: Model 1 was adjusted for age, sex, race, TDI score, education, and assessment center; Model 2 was further adjusted for health behaviors, including having moderate alcohol consumption, smoking status, regular physical activity, and a healthy diet, in addition to covariates in Model 1; based on Model 2, Model 3 was further adjusted for metabolic risk factors, including BMI, lipid-lowering medication, and blood pressure medication. In these models, moderate alcohol intake was defined as a maximum of one drink per day for women and a maximum of two drinks per day for males [31]; regular physical activity was defined as engaging in at least 150 min of moderate activity per week or 75 min of vigorous activity per week; a healthy diet was characterized as the consumption of a sufficient quantity of at least half of the ten recommended food categories [32, 33].

A series of subgroup analyses were performed to evaluate the potential effect modification by sex, age (< 60 years vs. ≥ 60 years), TDI score (above the median vs. below the median), and education (college or higher vs. less). In addition, to evaluate interactions between air pollution and the LE8, we first dichotomized the total LE8 score, as well as individual metrics, into two groups ("Low" and "High") according to their medians, and then explored associations of air pollutants across LE8 score groups. The significance of potential effect modification was assessed by including product terms of air pollutants and examined characteristics in the multivariable Cox models. A *P*-interaction < 0.1 was regarded as having suggestive interaction.

To further investigate the combined effect of LE8 and air pollution, we constructed a nine-level variable for each air pollutant-LE8 pair according to their tertiles (T1, "Low"; T2, "Moderate"; T3, "High") and modeled it as a categorical variable, with "Low air pollutant-High LE8" as a reference, in multivariable Cox regression adjusting for age, sex, race, TDI score, education, and assessment center.

Additionally, a set of sensitivity analyses was conducted. (1) Given that participants with T2D have a lower glucose score, we recalculated a modified LE8 by excluding the glucose score and repeated the combined effect analyses. (2) We additionally excluded participants with self-reported cancer and cardiovascular disease (Table S5) to estimate the effect of other diseases at baseline. (3) We excluded those who had developed severe NAFLD within two years of the air pollution evaluation to prevent the possibility of reverse causation. (4) Cox models with penalized splines (degree of freedom = 3) were employed to further evaluate the exposure–response relationship between air pollutants and severe NAFLD. (5) We treated death as a competing risk analysis using Fine-Gray subdistribution hazards regression models. (6) We recalculated the follow-up time from the date of the first diagnosis of T2D instead of baseline recruitment in our primary analyses.

All analyses were conducted in R (version 4.1.1). All statistical tests were two-sided. *P*-values < 0.05 were considered statistically significant.

## Results

Table 1 displays the baseline characteristics of individuals in the present study (*N* = 23,129). A total of 1,123 (4.9%) individuals were identified as having severe NAFLD over a median follow-up period of 13.6 years. Overall, participants had a mean (SD) age of 60 (6.9) years and were predominantly male (62.2%). Most of the participants were White (86.3%), less educated (77%), non-smokers (88.5%), had an unhealthy diet (79.5%), were less likely to exercise regularly, and drank moderate amounts of alcohol. Participants with severe NAFLD tended to be younger (59.2 years vs. 60 years), female (44.1% vs. 37.5%), and White (89% vs. 86.2%), had lower education levels (18.9% vs. 23.2%), and higher TDI score (0.01 vs. -0.53). In terms of health behavior, those with severe NAFLD were less likely to drink alcohol moderately (19% vs. 25.3%) and be involved in physical activity regularly (38.2% vs. 43.8%). As expected, severe NAFLD participants have unfavorable metabolic profiles and lower LE8 score (52.4 vs. 56.2). The median annual concentrations of PM_2.5_, PM_10_, PM_2.5–10_, NO_2_, and NOx were 10.1 μg/m^3^, 19.3 μg/m^3^, 6.2 μg/m^3^, 29.2 μg/m^3^, and 43.9 μg/m^3^ (Table S6), respectively. Those with severe NAFLD had higher exposure levels to these five air pollutants. Spearman correlation coefficients pertaining to different air pollutants are displayed in Table S6.

**Table 1:** Baseline characteristics of participants by the status of severe NAFLD

Variables	Total participants (*N* = 23,129)	Non-NAFLD (*N* = 22,006)	NAFLD (*N* = 1,123)
Population characteristics and lifestyle factors			
Age, mean (standard deviation [SD]), Years	60 (6.9)	60 (6.9)	59.2 (7.3)
Women, Yes, n (%)	8,745 (37.8%)	8,250 (37.5%)	495 (44.1%)
White, Yes, n (%)	19,969 (86.3%)	18,969 (86.2%)	1,000 (89%)
College/university degree, Yes, n (%)	5,316 (23%)	5,104 (23.2%)	212 (18.9%)
TDI score, mean (SD)	-0.5 (3.4)	-0.53 (3.4)	0.01 (3.4)
Moderate alcohol consumption, Yes, n (%)	5774 (25%)	5561 (25.3%)	213 (19%)
Smoking status, Never/Previous, n (%)	20466 (88.5%)	19478 (88.5%)	988 (88%)
Regular physical activity, Yes, n (%)	10076 (43.6%)	9647 (43.8%)	429 (38.2%)
Healthy diet, Yes, n (%)	4730 (20.5%)	4511 (20.5%)	219 (19.5%)
BMI, kg/m^2^	31.6 (5.8)	31.5 (5.8)	33.7 (5.9)
Blood pressure medication, Yes	13941 (60.3%)	13223 (60.1%)	718 (63.9%)
Lipid-lowering medication, Yes	16005 (69.2%)	15222 (69.2%)	783 (69.7%)
Mean total LE8 scores**,** mean (SD)	56 (11.9)	56.2 (11.9)	52.4 (11.8)
LE8 metrics, median (Q1, Q3)			
Tobacco/nicotine exposure score	50 (50, 100)	50 (50, 100)	50 (50, 100)
Physical activity score	90 (20, 100)	90 (20, 100)	80 (0, 100)
Diet score	25 (25, 50)	25 (25, 50)	25 (25, 50)
Sleep health score	100 (70, 100)	100 (70, 100)	100 (70, 100)
BMI score	30 (30, 70)	30 (30, 70)	30 (15, 70)
Blood lipid score	80 (40, 80)	80 (40, 80)	80 (40, 80)
Blood pressure score	30 (25, 50)	30 (25, 50)	30 (25, 50)
Glucose score	40 (30, 40)	40 (30, 40)	40 (30, 40)
Daily cumulative exposure, (μg/m^**3**^), median (Q1, Q3)			
PM_2.5_	10.1 (9.4, 10.7)	10.1 (9.4, 10.7)	10.2 (9.6, 10.8)
PM_10_	19.3 (18.3, 20.7)	19.3 (18.3, 20.7)	19.5 (18.4, 20.9)
PM_2.5–10_	6.2 (5.9, 6.7)	6.2 (5.9, 6.7)	6.2 (5.9, 6.8)
NO_2_	29.2 (24.1, 35.2)	29.1 (24.1, 35.1)	30.3 (25.5, 36.2)
NO_X_	43.9 (36.2, 52.4)	43.8 (36.2, 52.3)	45.3 (37.5, 54.1)

Values are the mean (± standard deviation, SD) or median (Q1, Q3) for continuous variables and n (percentage, %) for categorical variables; Q1 and Q3 refer to the 25^th^ and 75^th^ percentile.

As shown in Table 2, per IQR increase in concentrations of PM_2.5_ (hazard ratio [HR] = 1.12; 95% confidence interval [CI]: 1.04, 1.2), PM_2.5–10_ (1.1 [1.03, 1.18]), PM_10_ (1.11 [1.03, 1.2]), NO_2_ (1.13 [1.04, 1.23]), and NO_x_ (1.07 [1.01, 1.13]) were associated with an elevated risk of severe NAFLD in a linear dose–response manner (all *P*-trend < 0.05, Model 1). Using penalized spline in Cox models confirmed that the associations were almost linear without appreciable threshold (Fig. S2). These associations largely persisted with further adjustment of health behavior factors (Model 2) except for those of PM_2.5–10_ (Table 2). Furthermore, the associations of PM_2.5_ (1.12 [1.02, 1.23]), NO_2_ (1.15 [1.04, 1.27]), and NO_x_ (1.08 [1.01, 1.17]) with severe NAFLD remain significant with the further adjustment of NAFLD related metabolic traits and risk factors (Model 3) (Table 2), highlighting the independent effects of these air pollutants on the risk of severe NAFLD.

**Table 2:** Associations of ambient air pollution with severe NAFLD among participants with T2D

Air pollution	Quartile 1	Quartile 2	Quartile 3	Quartile 4	*P*-trend	Per IQR
PM_2.5_						
Events, N	226	279	294	324		1123
Model 1	Ref	1.21 (1.01, 1.44)	1.25 (1.05, 1.49)	1.27 (1.06, 1.53)	0.01	1.12 (1.04, 1.2)
Model 2	Ref	1.15 (0.95, 1.4)	1.27 (1.04, 1.54)	1.28 (1.04, 1.57)	0.01	1.11 (1.02, 1.2)
Model 3	Ref	1.25 (1.01, 1.56)	1.32 (1.06, 1.64)	1.37 (1.09, 1.72)	0.009	1.12 (1.02, 1.23)
PM _2.5–10_						
Events, N	241	295	292	295		1123
Model 1	Ref	1.23 (1.04, 1.46)	1.24 (1.04, 1.48)	1.24 (1.03, 1.49)	0.03	1.1 (1.03, 1.18)
Model 2	Ref	1.22 (1.01, 1.48)	1.24 (1.02, 1.51)	1.23 (1, 1.51)	0.06	1.08 (1, 1.17)
Model 3	Ref	1.29 (1.04, 1.58)	1.23 (0.99, 1.53)	1.2 (0.95, 1.51)	0.19	1.08 (0.99, 1.18)
PM _10_						
Events, N	253	261	291	318		1123
Model 1	Ref	1.01 (0.85, 1.2)	1.12 (0.95, 1.33)	1.24 (1.04, 1.49)	0.008	1.11 (1.03, 1.2)
Model 2	Ref	0.97 (0.8, 1.17)	1.13 (0.93, 1.36)	1.21 (0.99, 1.47)	0.02	1.1 (1.01, 1.21)
Model 3	Ref	0.95 (0.77, 1.18)	1.1 (0.9, 1.36)	1.14 (0.92, 1.42)	0.13	1.09 (0.99, 1.2)
NO_2_						
Events, N	222	285	306	310		1123
Model 1	Ref	1.25 (1.05, 1.49)	1.29 (1.08, 1.54)	1.28 (1.06, 1.56)	0.01	1.13 (1.04, 1.23)
Model 2	Ref	1.24 (1.02, 1.51)	1.27 (1.04, 1.55)	1.27 (1.02, 1.58)	0.03	1.13 (1.03, 1.23)
Model 3	Ref	1.31 (1.05, 1.62)	1.3 (1.04, 1.62)	1.3 (1.02, 1.65)	0.05	1.15 (1.04, 1.27)
NO_X_						
Events, N	239	262	301	321		1123
Model 1	Ref	1.08 (0.9, 1.29)	1.22 (1.02, 1.45)	1.2 (0.99, 1.44)	0.03	1.07 (1.01, 1.13)
Model 2	Ref	1.05 (0.87, 1.28)	1.21 (1, 1.47)	1.21 (0.98, 1.49)	0.04	1.07 (1, 1.14)
Model 3	Ref	1.05 (0.84, 1.3)	1.23 (0.99, 1.52)	1.21 (0.96, 1.53)	0.05	1.08 (1.01, 1.17)
Air pollution score						
Events, N	208	298	287	330		1123
Model 1	Ref	1.41 (1.18, 1.69)	1.33 (1.11, 1.6)	1.45 (1.2, 1.76)	0.001	1.03 (1.01, 1.05)
Model 2	Ref	1.45 (1.19, 1.76)	1.36 (1.11, 1.66)	1.5 (1.21, 1.87)	0.001	1.03 (1.01, 1.05)
Model 3	Ref	1.6 (1.28, 2)	1.47 (1.17, 1.85)	1.59 (1.24, 2.02)	0.001	1.03 (1.01, 1.05)

Model 1: adjusted for recruitment centers, age, sex, ethnicity, education, and TDI score; Model 2: Model 1 further adjusted for smoking, drinking, having regular physical activity, and healthy diet; Model 3: Model 2 further adjusted for BMI, lipid-lowering medication, blood pressure medication

By calculating a composite air pollution score consisting of the above five air pollutants, we observed that a higher air pollution score was associated with an elevated risk of severe NAFLD (1.03 [1.01, 1.05]) (Table 2, Model 1) with a significant linear trend (Fig. S2), which was robust to adjustments of health behaviors factors (1.03 [1.01, 1.05], Model 2) and NAFLD related metabolic traits or risk factors (1.03 [1.01, 1.05], Model 3) with a significant linear exposure–response trend (all *P*-trend < 0.05).

Subgroup analyses by various predefined factors are shown in Fig. S3. Overall, we did not find any significant effect modification by sex, age, TDI score, and education (Fig. S3).

Per point increase in LE8 score was negatively associated with the risk of severe NAFLD (HR = 0.97 [0.97, 0.98], per point increase). Compared with participants in the lowest tertile of LE8, those in the highest tertile of LE8 had a lower risk of severe NAFLD (0.47 [0.39, 0.57]).

In stratified analyses, we found positive but generally insignificant associations for air pollutants with severe NAFLD in each LE8 stratum (Table 3). In contrast, significant negative associations of LE8 with severe NAFLD were observed in each air pollutant stratum (Fig. S4). The overall interaction between air pollution and LE8 and most of its components was insignificant (Table 3, Fig. S3). The associations of PM_10_ with severe NAFLD were more pronounced among those who engaged in lower physical activity levels (*P*-int = 0.07, Fig. S3). The associations of NO_2_ and air pollution score with the risk of severe NAFLD were stronger among participants with a healthier diet (*P*-int = 0.02 and *P*-int = 0.06, respectively, Fig. S3). The associations of NO_x_ with severe NAFLD were more evident among participants with sufficient sleep (*P*-int = 0.06, Fig. S3).

**Table 3:** Association of long-term exposure to air pollutants (per IQR increase) and risk of severe NAFLD according to the LE8 scores

Exposure	low LE8	Moderate LE8	High LE8	*P*-int
PM_2.5_	1.09 (0.96, 1.23)	1.14 (0.97, 1.34)	0.99 (0.81, 1.22)	0.8
PM_2.5–10_	1.08 (0.96, 1.22)	1.05 (0.9, 1.23)	1.11 (0.92, 1.34)	0.42
PM_10_	1.11 (0.97, 1.28)	1.06 (0.89, 1.26)	0.97 (0.78, 1.21)	0.35
NO_2_	1.2 (1.04, 1.38)	1.11 (0.93, 1.32)	1.15 (0.93, 1.43)	0.56
NO_X_	1.04 (0.94, 1.16)	1.09 (0.96, 1.24)	0.98 (0.82, 1.17)	0.63
Air pollution score	1.03 (1.01, 1.07)	1.03 (0.99, 1.07)	1.03 (0.98, 1.08)	0.58

*P*-int, *P* values for interaction; estimates refer to per IQR increase in air pollutants; LE8 were categorized into "Low", "Moderate", and "High" according to their tertiles. A higher total LE8 score (range 0–100) reflects greater metabolic and behavioral health. Models were adjusted for recruitment centers, age, sex, ethnicity, education, and TDI score

**Fig. 1:**
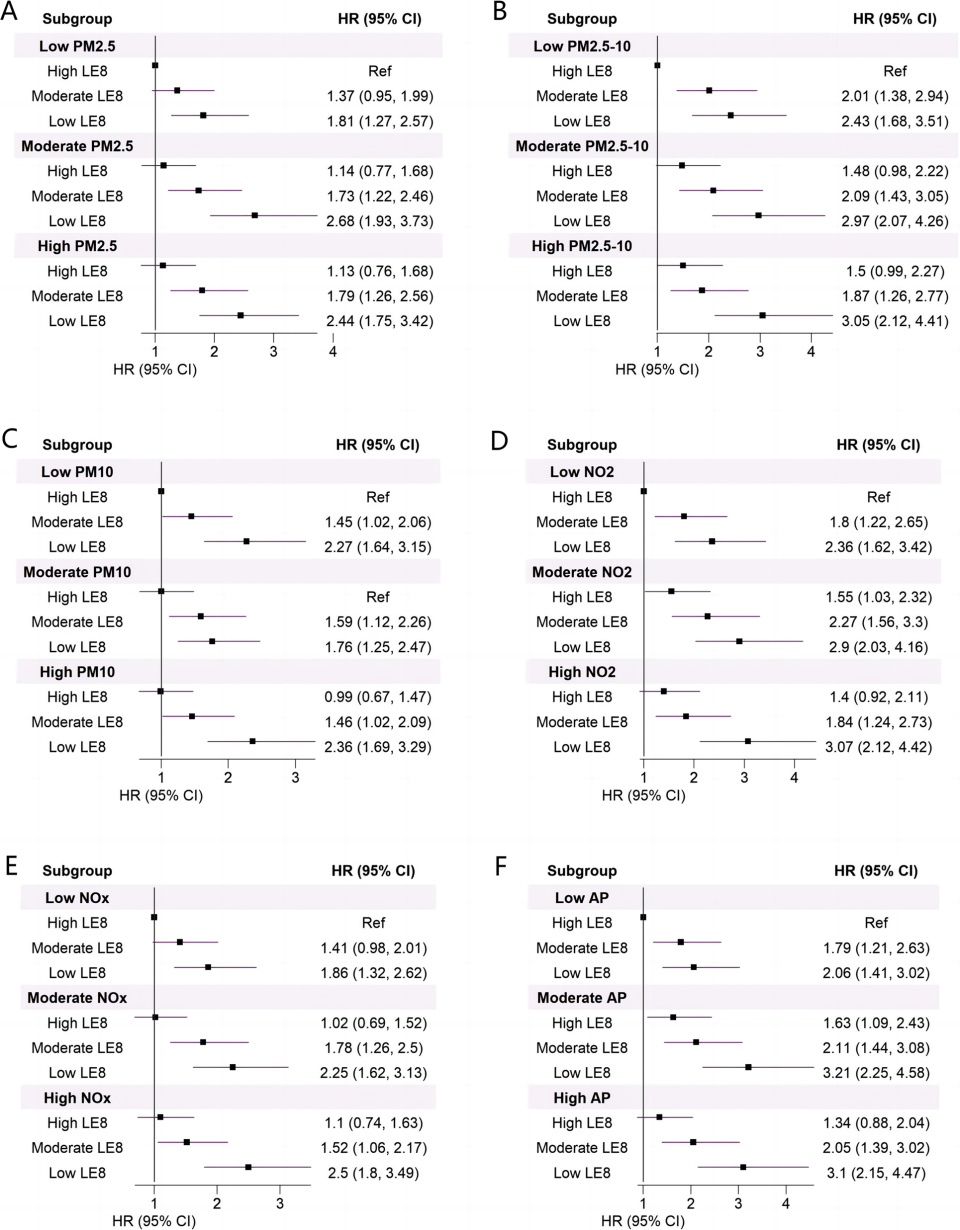
Combined effects of LE8 and air pollution on the risk of severe NAFLD among T2D participants. Both LE8 and pollution indexes were categorized into "Low", "Moderate", and "High" according to their tertiles. A higher LE8 score indicates healthier health-related behaviors and factors, whereas higher air pollutants reflect higher exposure levels. Models were adjusted for recruitment centers, age, sex, ethnicity, education, and TDI score; AP, air pollution score; Ref, reference

The combined effects of LE8 score and air pollutants on the risk of severe NAFLD behaved in a dose–response manner (Fig. 1). Our analysis revealed that individuals within the high-air pollutants and low-LE8 group had the highest risk of developing severe NAFLD (except for the high-PM_2.5_ and high-AP). In contrast to the participants in the low air pollution and high LE8 groups, the HRs (95%CI) for severe NAFLD among participants with low LE8 in the high PM_2.5_ group was 2.44 (1.75, 3.42), in the high PM_2.5–10_ group was 3.05 (2.12, 4.41), in the high PM_10_ group was 2.36 (1.69, 3.29), in the high NO_2_ group was 3.07 (2.12, 4.42), in the high NOx group was 2.5 (1.8, 3.49), and in the high air pollution score group was 3.1 (2.15, 4.47).

In sensitivity analyses, recalculated LE8 (excluding glucose score) did not change the combined effects of air pollution and LE8 on the risks of severe NAFLD (Fig. S5). There was no significant alteration in the association between each air pollutant and severe NAFLD when the individuals with self-reported cancer diseases and cardiovascular disease (Table S7, Model 1) or those with a follow-up period of less than two years were excluded from the analysis (Table S7, Model 2). The results were also robust in the competing risk model (Table S7, Model 3), and the models using the recalculated the follow-up time from the date of the first diagnosis of T2D instead of baseline recruitment (Table S7, Model 4).

## Discussion

In this large prospective cohort study, higher levels of PM_2.5_, NO_2_, NOx, and air pollution score were associated with elevated risk of severe NAFLD among participants with T2D. In addition, individuals with higher levels of air pollutants (i.e., PM_2.5_, PM_2.5–10_, PM_10_, NO_2_, and NO_X_,) and lower LE8 score, had higher risks of severe NAFLD as compared to those with low levels of air pollutants and high LE8 score. Although the effect modifications by LE8 were non-significant, our findings highlighted that a healthier LE8 score might attenuate the elevated risk of severe NAFLD pertinent to air pollution exposure, contributing to promoting potential health policies tailored to combat the negative impacts of air pollution.

Diabetic status was shown to have the potential to modify the associations between air pollution and hypertension [34], cardiovascular diseases (CVD) [35], and chronic obstructive pulmonary disease [17]. Similar evidence of exacerbated detrimental impacts of air pollution on pathogenesis involved in NAFLD, such as oxidative stress induction, inflammatory activation, and insulin resistance, in the context of diabetes has been reported in experimental studies [9]. Given the pivotal role of T2D in the etiology of NAFLD [8], these observations collectively indicate the necessity of reducing the risk of diabetes and the overall metabolic disorder to reduce further the impact of air pollution in general and susceptible populations.

Our findings provided epidemiological evidence supporting the association of long-term exposure to particulate matter (i.e., PM_2.5_) and nitrogen oxides (i.e., NO_2_, NOx) with the increased risk of severe NAFLD among participants with T2D. Experimental studies suggested that PM_2.5_ exposure could impair glucose homeostasis [36] and promote hepatic fibrogenesis [37], both of which are known to play significant roles in the pathways associated with the progression of NAFLD [38]. Four epidemiological studies have been conducted to investigate the potential relationship between air pollution and NAFLD, and two have explored the modification effect of diabetes [11–14]. For example, a cross-sectional study involving 90,086 Chinese adults found that higher levels of PM_2.5_, PM_10_, and NO_2_ were associated with the elevated prevalence of metabolic dysfunction-associated fatty liver disease among participants with diabetes [12]. Another cross-sectional study conducted among US hospitalized patients found that PM_2.5_ exposure was positively associated with prevalent NAFLD in participants with diabetes [14]. However, no prior longitudinal studies have examined the correlations between air pollution and the subsequent risk of NAFLD in individuals with T2D.

Our study provided a significant implication on the potential interaction between LE8 and air pollution in relation to the risk of severe NAFLD. As an index originally proposed to quantify overall cardiometabolic health, LE8 has been closely linked to NAFLD [21]. The present study, for the first time, assessed the joint associations of LE8 and each air pollutant with the risk of NAFLD. When exploring the associations between air pollution and severe NAFLD across individual LE8 metrics, our findings highlighted the effect modification by physical activity, with attenuated associations of PM_10_ with severe NAFLD found among groups with high physical activity score. This finding aligns with other studies investigating the effect of modification of physical activity in the relationship between air pollution and CVD risk, in which regular physical exercise may safeguard against the detrimental cardiovascular consequences of air pollution [39, 40]. Nevertheless, some studies have shown that the positive associations of air pollution with stroke and elevated blood pressure were particularly notable among those engaging in high levels of physical activity [41, 42]. The observed disparity may be explained by variations in air pollution levels among individuals with different levels of physical activity, indicating that the beneficial effects of physical exercise may be attenuated in extreme levels of air pollution exposure.

We also found that the association between NO_2_ and severe NAFLD was stronger among participants with healthier diets, especially those who consume more vegetables and fruit but less processed meat, refined grain, and sugar. This finding is unexpected and in contrast to the known beneficial effects of a healthier diet. Further replications are needed to determine whether the present observations are results by chance. Another unexpected result was that the association of NO_X_ with the risks of severe NAFLD was more evident among participants with sufficient sleep. As a recently added CVH metric, sleep health demonstrated a positive association with NAFLD [21]. One plausible explanation is that adequate sleep duration is positively associated with extended outdoor activities, potentially increasing the duration of exposure to ambient air pollution. This extended exposure could, in part, contribute to the observed risk associated with NOx exposure in individuals who obtain sufficient sleep. Nevertheless, further investigation is warranted to validate these results and explore the underlying mechanism.

Our study has several limitations. First, air pollution exposure and LE8 were not assessed during the follow-up, limiting the capacity to determine the effects of longitudinal dynamic change of both air pollutants and LE8 on NAFLD. Second, the current study used hospital inpatient data and death registry records to ascertain the outcome, which may be restricted to more advanced or severe NAFLD. Consequently, there is a possibility that asymptomatic NAFLD cases may have been overlooked, and the potential health risks associated with air pollution may have been underestimated. Third, the studied population was from the UK, where air pollution levels are quite low; the observed associations should be interpreted cautiously when extrapolated to areas with comparatively higher air pollution levels. Finally, although we have thoroughly considered typical risk factors and potential confounding variables, residual confounding cannot be fully excluded.

## Conclusions

In this large population-based cohort study, we found that long-term exposure to ambient PM_2.5_, NO_2_, and NOx was associated with an increased risk of severe NAFLD among individuals with T2D. Our results indicated that individuals with higher air pollution levels but lower LE8 score tended to have higher risks of NAFLD than those with low air pollution exposure levels and high LE8 score. Due to the observational nature of the present study, our main findings need to be validated in experimental or intervention settings to assess the causality.

### Supplementary Information

**Supplementary Material 1. **

## Data Availability

Data are accessible with license from the UK Biobank. Detailed Information about how to acquire access to UK Biobank can be found at https://www.ukbiobank.ac.uk/.

## Abbreviations

LE8: Life’s Essential 8
NAFLD: Non-alcoholic fatty liver disease
NASH: Non-alcoholic steatohepatitis
NO_2_: Nitrogen dioxide
NO_X_: Nitrogen oxides
PM: Particulate matter
T2D: Type 2 diabetes
