# Supplementary material for: Air pollution, life’s essential 8, and risk of severe non-alcoholic fatty liver disease among individuals with type 2 diabetes
Source: BMC Public Health. 2024 May 20;24:1350. doi: 10.1186/s12889-024-18641-4 (PMC11103844; doi:10.1186/s12889-024-18641-4)
Supplement: Supplementary file 1 — Supplementary Material 1. [file 12889_2024_18641_MOESM1_ESM.docx]

**Supplementary Materials**

**Air Pollution, Life’s Essential 8, and Risk of Severe Non-Alcoholic Fatty Liver Disease Among Individuals with Type 2 Diabetes**

Ruxianguli Aimuzi^1^, Zhilan Xie^1^, Yimin Qu^1^, Yu Jiang^1*^

^1^ School of Population Medicine and Public Health, Chinese Academy of Medical Sciences and Peking Union Medical College, Beijing, 100730, China

*Correspondence to: Dr. Yu Jiang, School of Population Medicine and Public Health, Chinese Academy of Medical Sciences and Peking Union Medical College; E-mail address: [jiangyu@pumc.edu.cn](mailto:jiangyu@pumc.edu.cn).

**Legends of supplementary figures and tables**

Fig. S1. Flowchart of the study population.

Fig. S2. Association between air pollution and risk of severe NAFLD among participants with T2D.

Fig. S3. Associations of air pollution with severe NAFLD, stratified by potential modifiers.

Fig. S4. Associations of total LE8 score with severe NAFLD stratified by air pollutants.

Fig. S5. Combined effects of Life’s Essential 8 (LE8) *, air pollution and the risk of severe NAFLD

Table S1. Codes used in the UK Biobank study to identify prevalent diseases.

Table S2. International Classification of Diseases (ICD) codes used in the UK Biobank to identify NAFLD.

Table S3. Methods for evaluating each Life’s Essential 8 metric

Table S4. Methods for assessing healthy diet score in the present study

Table S5. Codes used in the UK Biobank study to identify baseline cancer and cardiovascular disease.

Table S6. Descriptive statistics of pollutants and correlation matrix.

Table S7. Sensitivity Analysis of the effect of ambient air pollution on the risks of severe NAFLD.


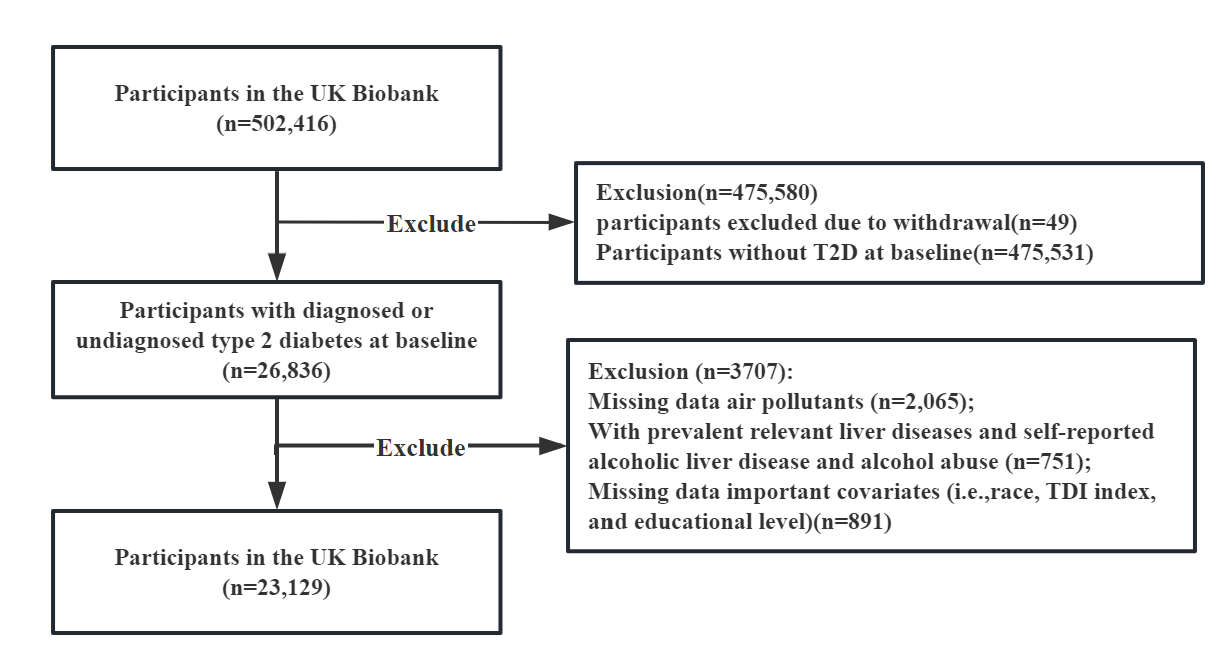


**Fig. S1. Flowchart of the study population.**


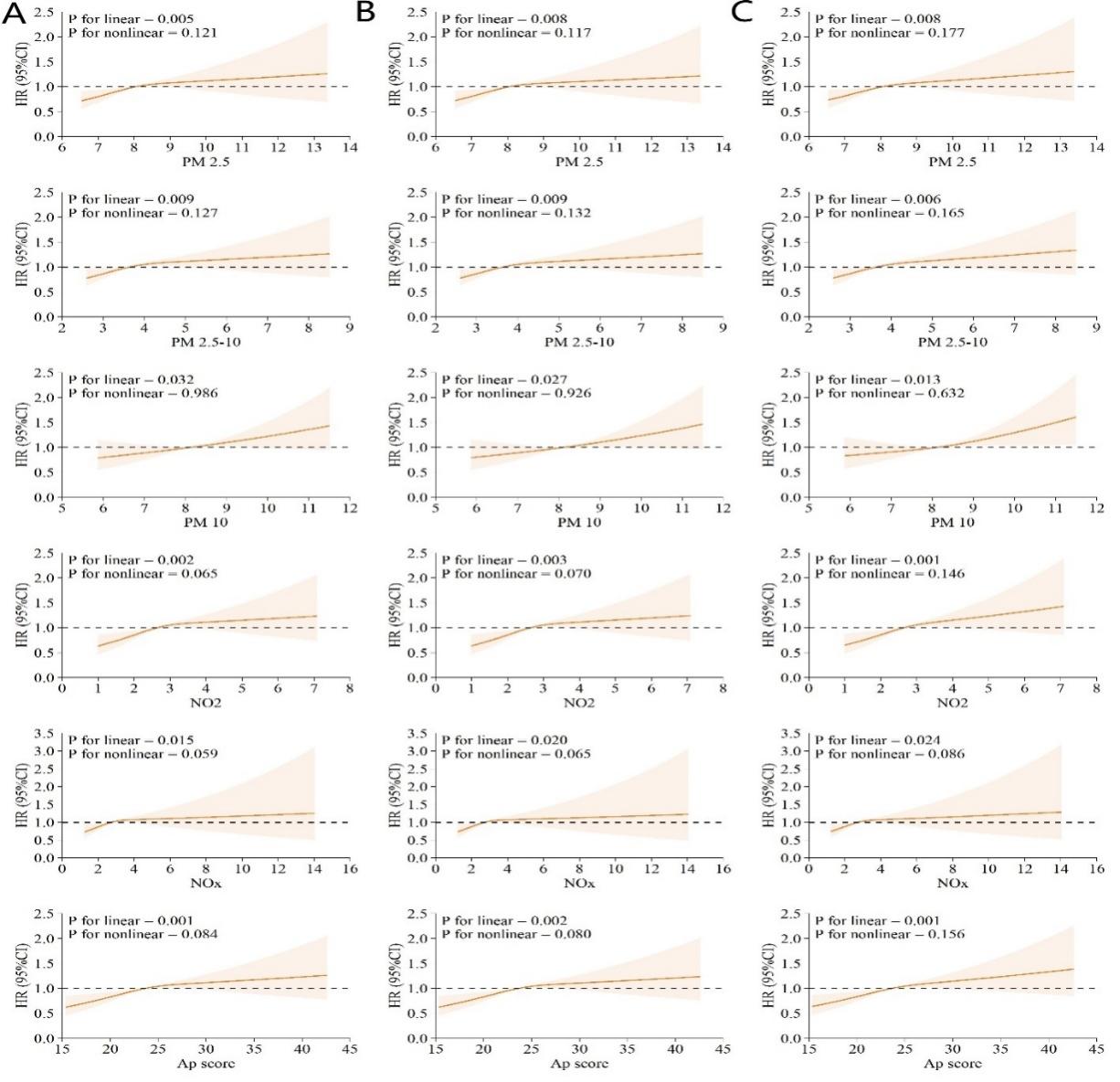


**Fig. S2. Association between air pollution and incident severe NAFLD among participants with T2D.** A penalized spline smoothing function (degree of freedom=3) was used to assess the concentration-response relation. **A (Model 1):** adjusted for recruitment centers, age, sex, ethnicity, education, and TDI score; **B (Model 2):** Model 1 further adjusted for smoking, drinking, having regular physical activity, and healthy diet; **C (Model 3):** Model 2 further adjusted for BMI, lipid-lowering medication, blood pressure medication


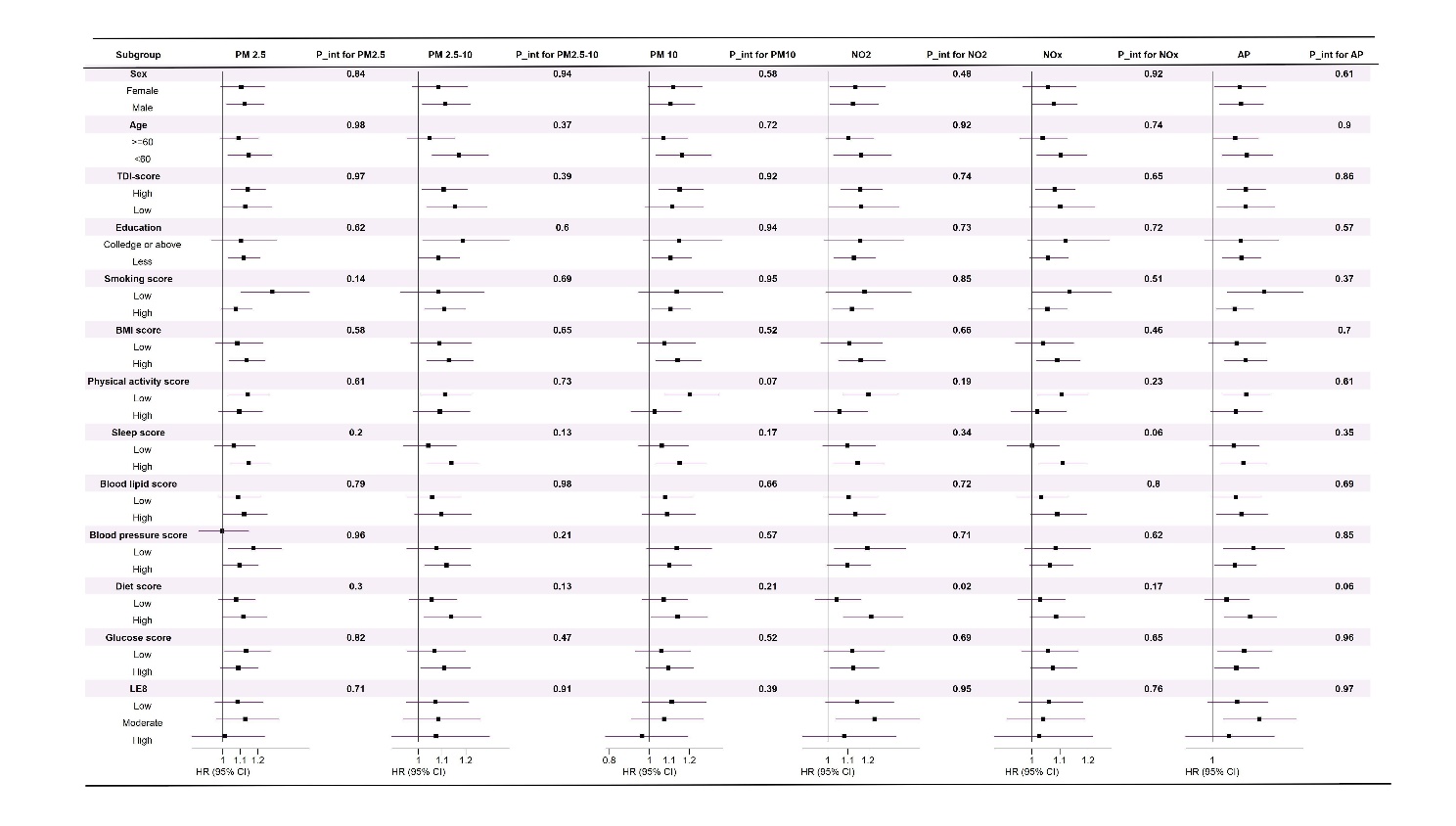
**Fig. S3. Associations of air pollution with severe NAFLD, stratified by potential modifiers.** Estimates refer to per IQR increment in air pollutants. TDI score were categorized into (“Low” and “High”) according to median; smoking score, BMI score, Physical activity score, Sleep score, Blood lipid score, Blood pressure score, and Diet score were categorized into ("Low" and "High") according to their medians. LE8 were classified into “Low”, “Moderate”, and “High” according to their tertiles. Cox regression models were applied. Models were adjusted for age, sex, race, education, TDI score, and recruitment centers. AP, air pollution; *P*-int, P value for interaction.


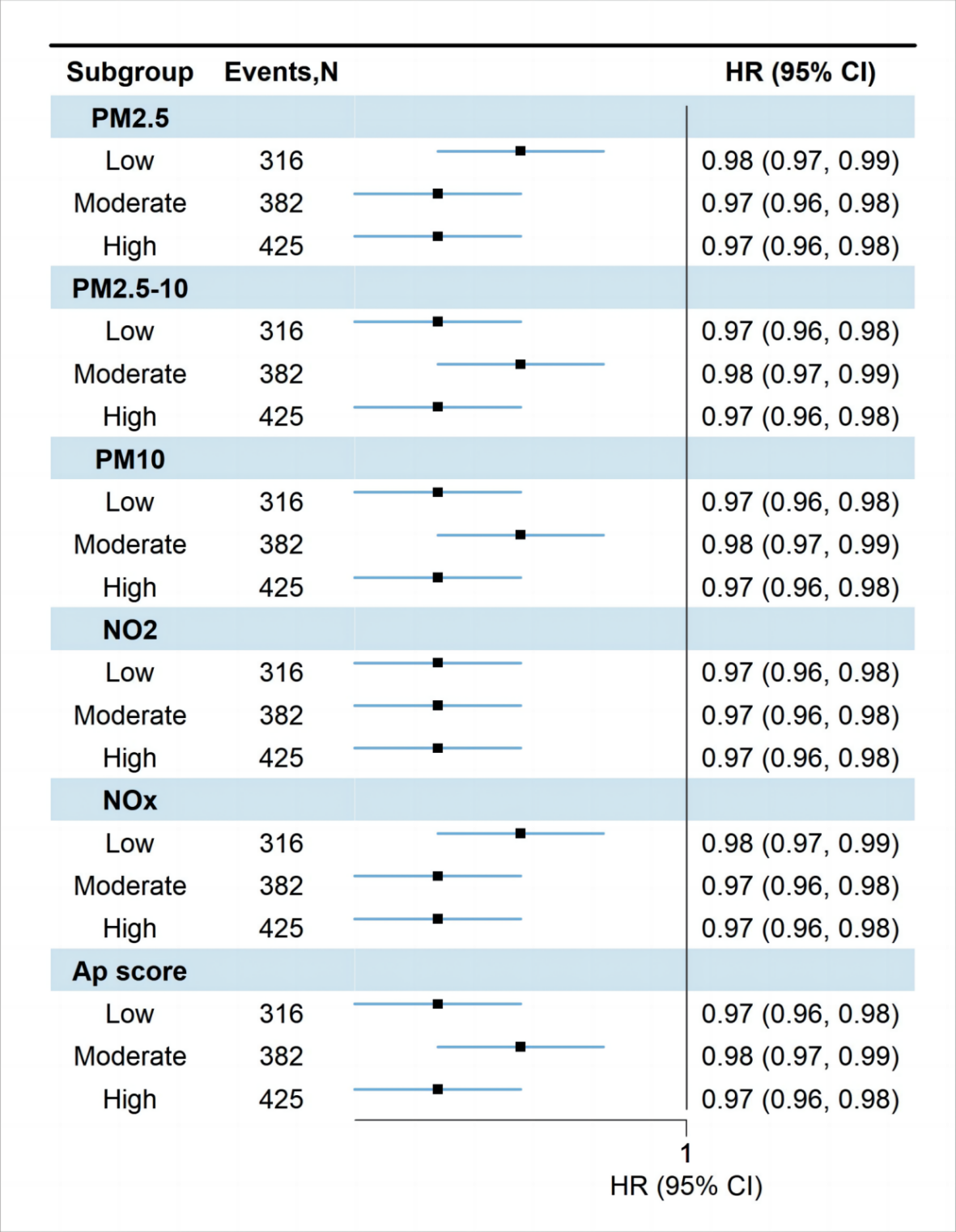
**Fig. S4. Associations of total LE8 score with severe NAFLD stratified by air pollutants.** Estimates refer to per point increment in LE8. Air pollutants were categorized into "Low", "Moderate", and "High," according to their tertiles. Cox regression models were applied. Models were adjusted for recruitment centers, age, sex, ethnicity, education, and TDI score. Ap, air pollution.


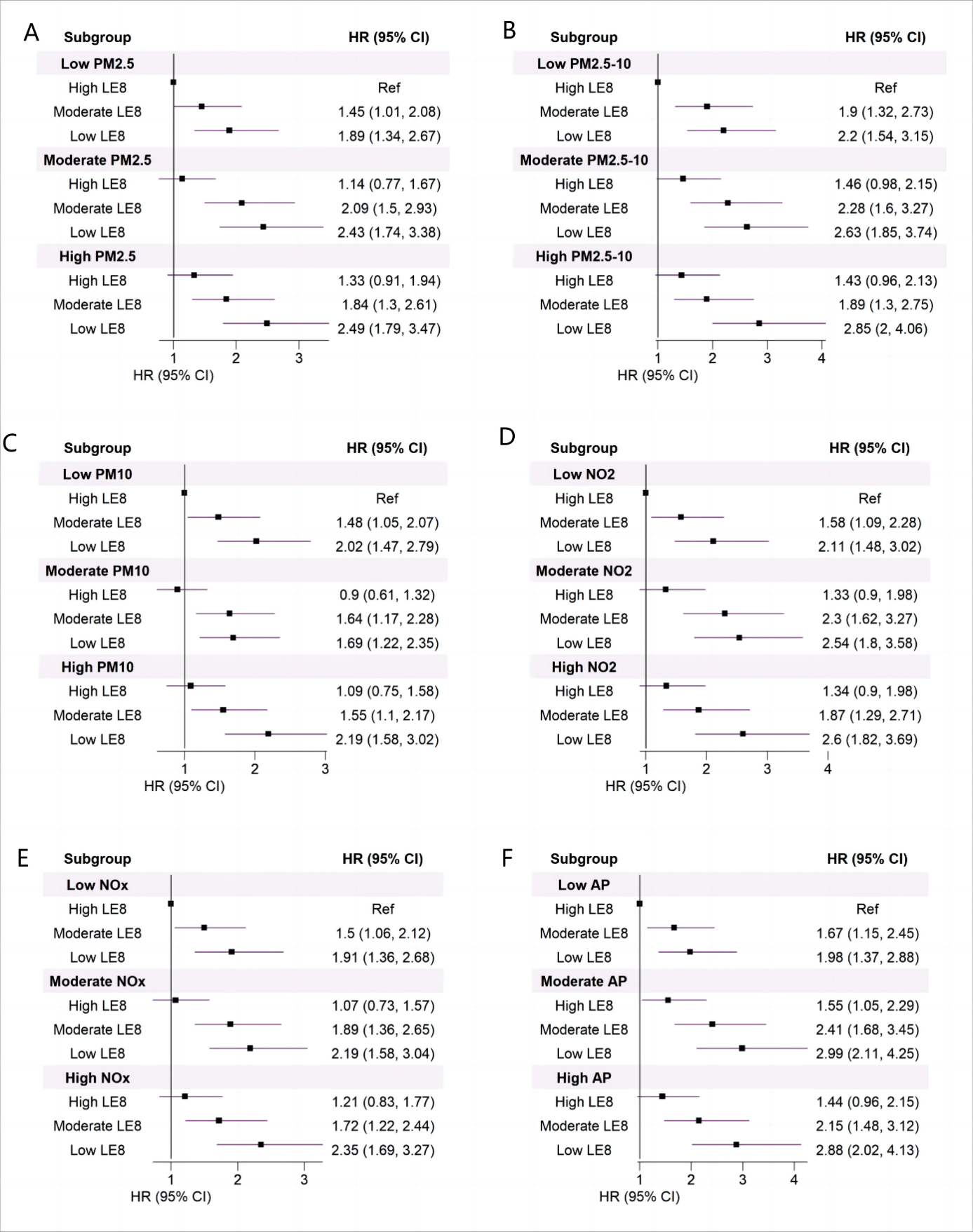


**Fig. S5. Combined effects of Life’s Essential 8 (LE8) *, air pollution, and the risk of severe NAFLD.**

Note: *LE8 was recalculated by excluding glucose score. Both LE8 and pollution were categorized into "Low", "Moderate", and "High" according to their tertiles. Cox regression models were applied. Models were adjusted for recruitment centers, age, sex, ethnicity, education, and TDI score.

**Table S1. Codes used in the UK Biobank study to identify prevalent diseases.**

| Prevalent diseases | Self-reported | ICD-9 | ICD-10 |
| --- | --- | --- | --- |
| **Severe liver diseases (**Compensated cirrhosis, decompensated cirrhosis, liver failure, hepatocellular carcinoma, liver transplant status) | - | 571.5, 456.1, 456.21, 456.0, 456.20, 789.5, 572.2, 572.4, 572.3, 572.8, 570, 155.0, V42.7 | K74.1, K74.2, K74.6, I85.9, I98.2, I86.4, I85.0, I98.3, R18, K76.7, K76.6, K72.1, K72.9, K72.0, C22.0, Z94.4 |
| **Other liver diseases at/before baseline （**ALD, Viral Hepatitis, Autoimmune liver disease, Hemochromatosis, Wilson, Alpha-1-antitrypsin deficiency, Budd-Chiari, Chronic hepatitis, unspecified, Secondary or unspecified biliary cirrhosis, Nonspecific reactive hepatitis, Toxic liver disease） | 1604, 1578, 1579, 1580, 1581, 1582, 1156, 1475, 1158, 1506, 1507, 1496 | 571.0-571.3, 070, 571.6, 576.1, 275.0, 275.1, 277.6,  453.0, 571.4, 571.6 | K70, B15, B16, B17, B18, B19, K83.0, K74.3, K75.4, E83.1, E.83.0, E88.0, I82.0, K76.5, K73.9, K73.2, K74.4, K74.5, K75.2, K71 |
| **Alcohol/drug use disorders at/before baseline （**alcohol use disorders, a somatic consequence of alcohol, drug use disorders except nicotine/caffeine | 1408, 1409 | 303, 305.0, 291, 357.5, 425.5, 535.3, 980.1, 980.9, 305.1-305.9 | F10, E24.4, G62.1, I42.6, K29.2, G31.2, G72.1, K85.2, K86.0, T51.0, T51.9, X65, Y57.3, Z50.2, Z71.4, Z72.1, F11-F14, F16, F18, F19 |

Abbreviation: ICD, International Classification of Diseases.

**Table S2. International Classification of Diseases (ICD) codes used in the UK Biobank to identify NAFLD.**

| severe NAFLD | ICD10 |
| --- | --- |
| Other specified inflammatory liver diseases | K758 |
| Fatty (change of) liver, not elsewhere classified | K760 |

Table S3. Methods for evaluating each Life’s Essential 8 metric

| Tobacco/nicotine exposure score | Self-reported tobacco use (current smoking status and history of smoking) or secondhand smoke exposure (Participants were asked, “Does anyone in your household smoke.” Secondhand smoke exposure was defined as if participants’ responses were “Yes, one household member smokes” or “Yes, more than one household member smokes”). | Points | Status |
| --- | --- | --- | --- |
|  |  | 100 | Never smoker |
|  |  | 75 | Former smoker quit ≥ 5 years. |
|  |  | 50 | Former smoker quit 1–<5 years. |
|  |  | 25 | Former smoker quit <1 year. |
|  |  | 0 | Current smoker |
|  | Subtract 20 points (unless the score is 0) for living with an active indoor smoker in the home. Moreover, the information on the specific time to quit smoking was only available to participants who indicated they smoked on most or all days in the past. We regard the participants who stated smoking occasionally as equivalent to Former smokers who quit 1–5 years.” We regard the participants who indicated just tried once or twice in the past” as equivalent to Former smokers who quit ≥ 5 years. | | |
| BMI | Body mass index (BMI) (calculated as weight (kg) divided by height in meters squared (m^2^) ). | 100 | <25 |
|  |  | 70 | 25–29.9 |
|  |  | 30 | 30–34.9 |
|  |  | 15 | 35–39.9 |
|  |  | 0 | ≥40 |
| Physical activity score | Self-reported minutes of moderate or vigorous physical activity per week. One minute of vigorous physical activity equals 2 minutes of moderate physical activity. | 100 | ≥150 |
|  |  | 90 | 120–149 |
|  |  | 80 | 90–119 |
|  |  | 60 | 60–89 |
|  |  | 40 | 30–59 |
|  |  | 20 | 1–29 |
|  |  | 0 | 0 |
| Diet | According to previous studies(Han et al., 2022; Said et al., 2018), we selected ten food groups, including fruits, vegetables, whole grains, (shell) fish, dairy, vegetable oils, refined grains, processed meats, unprocessed meat, and sugar-sweetened beverages, using Food Frequency Questionnaire (FFQ) (category 100052) from the UK Biobank. 1: If scoring condition met 0: If scoring condition not met (Range: 0-10) (See Table S4 for details) | 100 | 8-10 |
|  |  | 80 | 6-7 |
|  |  | 50 | 4-5 |
|  |  | 25 | 2-3 |
|  |  | 0 | 0-1 |
| Blood lipid score | Non-HDL cholesterol was calculated by total cholesterol minus HDL cholesterol. Serum cholesterol was measured enzymatically | 100 | <130 |
|  |  | 60 | 130–159 |
|  |  | 40 | 160–189 |
|  |  | 20 | 190–219 |
|  |  | 0 | ≥220 |
|  | If the drug-treated level, subtract 20 points (unless the score is 0) | | |
| Blood pressure score | The average of all available BP measurements was used to calculate systolic and diastolic BP. | 100 | <120/<80 (optimal) |
|  |  | 75 | 120–129/<80 (elevated) |
|  |  | 50 | 130–139 or 80–89 (stage 1 hypertension) |
|  |  | 25 | 140–159 or 90–99 |
|  |  | 0 | ≥160 or ≥100 |
|  | If the drug-treated level, subtract 20 points (unless the score is 0) | | |
| Glucose score | HbA1c was measured by high-performance liquid chromatography methods. | 100 | No history of diabetes and HbA1c <5.7 |
|  |  | 60 | No diabetes and HbA1c 5.7–6.4 (prediabetes) |
|  |  | 40 | diabetes with HbA1c <7 |
|  |  | 30 | diabetes with HbA1c 7–7.9 |
|  |  | 20 | diabetes with HbA1c 8–8.9 |
|  |  | 10 | diabetes with Hb A1c 9–9.9 |
|  |  | 0 | diabetes with HbA1c ≥10.0 |
| Sleep health score | Self-reported average hours of sleep per night | 100 | 7–<9 |
|  |  | 90 | 9–<10 |
|  |  | 70 | 6–<7 |
|  |  | 40 | 5–<6 or ≥10 |
|  |  | 20 | 4–<5 |
|  |  | 0 | <4 |

Table S4. Methods for evaluating healthy diet score in the present study

| Food groups | Components | Coding | Intake Goal |
| --- | --- | --- | --- |
| Vegetables | Cooked vegetables | ‘Less than once a week’=0.5; Amount per serving: cooked/raw vegetables – 3 heaped tablespoons | ≥ 3 servings/day |
|  | Salad/raw vegetables |  |  |
| Fruit | Fresh fruit | ‘Less than once a week’=0.5; Amount per serving: fresh fruit – 1piece; dried fruit – 5 pieces | ≥ 3 servings/day |
|  | Dried fruit |  |  |
| Fish | Oily fish | ‘Never’=0, ‘Less than once a week’=0.5, ‘Once a week’=1, ‘2-4 times a week’=3, ‘5-6 times a week’=5.5, ‘Once or more daily’=7 | ≥2 servings/week |
|  | Non-oily fish |  |  |
| Unprocessed meat | Beef |  | ≤ 2 servings/week |
|  | Pork |  |  |
|  | Lamb/mutton |  |  |
| Processed meat | Processed meat |  | ≤ 1 serving/week |
| Whole grains | Bread; Bread type  Cereal; Cereal type | Whole meal/whole grain bread - 1 slice  bran/oat/muesli cereal - 1 bowl | ≥ 3servings/day |
| Refined grains | Bread; Bread type  Cereal; Cereal type | white, brown, or other bread- 1 slice  biscuit, other- 1 bowl | ≤2 servings/day |
| Dairy | Cheese  Milk | Cheese - 1 piece/day  Milk - 1 glass/day | ≥ 2 servings/day |
| Vegetable oils | Spread  Bread type | Flora Pro-Active/Benecol spread  Flora Pro-Active/Benecol, soft margarine -, olive oil based -, polyunsaturated/sunflower based oil-based -, other low/reduced fat spread  Bread - 2 slices | ≥ 2 servings/day |
| Sugar-sweetened beverages | Sugar-sweetened beverages | 6144-"Which of the following do you NEVER eat?" | Don't drink |

Note: 1: If the scoring condition met the intake goal, 0: If the scoring condition did not meet the intake goal (Range: 0-10)

**Table S5. Codes used in the UK Biobank study to identify baseline cancer and cardiovascular disease.**

| Prevalent diseases | Self-reported | ICD-9 | ICD-10 |
| --- | --- | --- | --- |
| Cancer | 2453, 20001 |  |  |
| Cardiovascular Disease (CVD) | 6150 (1, 2, 3), 20002  (1074, 1075, 1081, 1583, 1086, 1491) | 410-414, 430-434, 436 | I20-I25, I60-I64 |

**Table S6. Descriptive statistics of pollutants and correlation matrix.**

| Pollution | Mean (SD) (μg/m^3^) | Minimum (μg/m^3^) | Maximum (μg/m^3^) | IQR  (μg/m^3^) | Spearman correlation coefficient | | | | |
| --- | --- | --- | --- | --- | --- | --- | --- | --- | --- |
|  |  |  |  |  | PM_2.5_ | PM_10_ | PM_2.5–10_ | NO_2_ | NOx |
| PM_2.5_ | 10.14 (1.07) | 8.17 | 19.65 | 1.25 | 1 | 0.6** | 0.29** | 0.72** | 0.86** |
| PM_10_ | 19.57 (1.96) | 13.53 | 28.41 | 2.39 |  | 1 | 0.59** | 0.74** | 0.63** |
| PM_2.5–10_ | 6.47 (0.9) | 5.57 | 12.82 | 0.83 |  |  | 1 | 0.29** | 0.3** |
| NO_2_ | 30.67 (9.39) | 9.19 | 95.43 | 11.02 |  |  |  | 1 | 0.79** |
| NOx | 46.31 (16.59) | 19.74 | 255.33 | 16.18 |  |  |  |  | 1 |

Note: Spearman correlation was applied, ** *P* < 0.01.

Table S7. Sensitivity Analysis of the effect of ambient air pollution on the risks of severe NAFLD.

| Pollution | Model 1 | Model 2 | Model 3 | Model 4 |
| --- | --- | --- | --- | --- |
| PM_2.5_ | 1.15 (1.05, 1.25) | 1.13 (1.05, 1.22) | 1.11 (1.03, 1.19) | 1.12 (1.04, 1.2) |
| PM_10_ | 1.16 (1.05, 1.27) | 1.1 (1.02, 1.2) | 1.11 (1.02, 1.2) | 1.12 (1.03, 1.21) |
| PM_2.5–10_ | 1.16 (1.07, 1.26) | 1.1 (1.02, 1.18) | 1.11 (1.04, 1.19) | 1.1 (1.03, 1.18) |
| NO_2_ | 1.17 (1.06, 1.29) | 1.14 (1.05, 1.24) | 1.14 (1.05, 1.23) | 1.14 (1.05, 1.23) |
| NOx | 1.08 (1.01, 1.16) | 1.08 (1.02, 1.14) | 1.07 (1.01, 1.14) | 1.07 (1.02, 1.13) |
| AP score | 1.04 (1.02, 1.06) | 1.03 (1.01, 1.05) | 1.03 (1.01, 1.05) | 1.03 (1.01, 1.05) |

Note: Estimates refer to per IQR increment in each air pollutant. AP, air pollution.

Model 1: Exclude the participants with prevalent cancer and CVD; Cox regression models were applied, and models were adjusted for UK Biobank assessment center, age, sex, ethnicity, education, and TDI score.

Model 2: Exclude the participants with follow-up years less than two years; Cox regression models were applied, and models were adjusted for the UK Biobank assessment center, age, sex, ethnicity, education, and TDI score.

Model 3: We treated death as a competing risk instead of censor in our primary analysis using Fine-Gray subdistribution hazards regression models, and models were adjusted for the UK Biobank assessment center, age, sex, ethnicity, education, and TDI score.

Model 4: Follow-up time was reported from the first diagnosis of T2D to censor event date (i.e., death, hospitalization, or loss to follow-up); Cox regression models were applied, and models were adjusted for UK Biobank assessment center, age, sex, ethnicity, education, and TDI score.

Han, H., et al., 2022. Association of a Healthy Lifestyle With All-Cause and Cause-Specific Mortality Among Individuals With Type 2 Diabetes: A Prospective Study in UK Biobank. Diabetes Care. 45**,** 319-329.

Said, M. A., et al., 2018. Associations of Combined Genetic and Lifestyle Risks With Incident Cardiovascular Disease and Diabetes in the UK Biobank Study. JAMA Cardiol. 3**,** 693-702.
